# CS^2^-Collector: A New Approach for Data Collection in Wireless Sensor Networks Based on Two-Dimensional Compressive Sensing

**DOI:** 10.3390/s16081318

**Published:** 2016-08-19

**Authors:** Yong Wang, Zhuoshi Yang, Jianpei Zhang, Feng Li, Hongkai Wen, Yiran Shen

**Affiliations:** 1 College of Computer Science and Technology, Harbin Engineering University, Harbin 150001, China; wangyongcs@hrbeu.edu.cn (Y.W.); yangzhuoshi@hrbeu.edu.cn (Z.Y.); zhangjianpei@hrbeu.edu.cn (J.Z.); 2 School of Computer Science and Technology, Shandong University, Jinan 250100, China; fli@sdu.edu.cn; 3 Department of Computer Science, University of Oxford, Oxford OX1 3QD, UK; hongkai.wen@cs.ox.ac.uk

**Keywords:** two-dimensional compressive sensing, Kronecker product, wireless sensor networks

## Abstract

In this paper, we consider the problem of reconstructing the temporal and spatial profile of some physical phenomena monitored by large-scale Wireless Sensor Networks (WSNs) in an energy efficient manner. Compressive sensing is one of the popular choices to reduce the energy consumption of the data collection in WSNs. The existing solutions only consider sparsity of sensors’ data from either temporal or spatial dimensions. In this paper, we propose a novel data collection strategy, CS^2^-collector, for WSNs based on the theory of Two Dimensional Compressive Sensing (2DCS). It exploits both temporal and spatial sparsity, i.e., 2D-sparsity of WSNs and achieves significant gain on the tradeoff between the compression ratio and reconstruction accuracy as the numerical simulations and evaluations on different types of sensors’ data. More intuitively, with the same given energy budget, CS^2^-collector provides significantly more accurate reconstruction of the profile of the physical phenomena that are temporal-spatially sparse.

## 1. Introduction

The recent technological evolution of sensing devices has significantly broadened the applications of Wireless Sensor Networks (WSNs). However, the current implementations of WSNs are struggling with issues on the conflicts between the high accuracy requirement of recovering the profile of physical phenomena and the restricted resource constraints (energy and computation) of the embedded sensors. Specifically, in a real WSN deployment, especially for large-scale scenarios, the embedded and low cost sensors periodically sample, send and relay data to a base station. The energy consumption of each sensor normally consists of sensors’ sampling, radio communication, microcontroller and quiescent consumption. Among all the factors of energy consumption, the communication costs take a substantial share of total energy consumption. For example, Table 1 shows the energy load of a typical humidity sensor in WSNs that were evaluated in [1]. The statistics demonstrate that the radio communication part consumes approximately 86% of the entire energy. Therefore, reducing the energy consumption of data communication is the key to extending the lifespan of WSNs.

Recent research has proposed data collection strategies based on compression methods in WSNs to reduce the overall amount of data transmitted through the network to effectively save the energy of each sensor so as to improve the lifespan of the whole WSNs [2,3]. The emerging mathematical theory of Compressive Sensing (CS) [4,5] has triggered the possibility to further minimize communication cost by compressing the data locally on the embedded sensors. With the same target of reconstruction accuracy at the base station, CS provides promising performance on reducing the sampling and communication cost [6,7,8]. CS can be applied to significantly reduce the dimensionality of the signals while preserving most of the information as far as the signals can be sparsely represented in some transform domain, such as Discrete Cosine Transform (DCT) and wavelet domains [9,10,11]. Intuitively, if a signal can be sparsely represented in some transform basis or dictionary, most of the information can be compressed within a significantly less number of random projections, and the original signal can be reconstructed accurately with a high probability via a number of different $\ell_{1}$ optimization algorithms.

Traditional data collection approaches sample signals at a frequency of at least Nyquist rate (i.e., twice of the highest frequency of the original signal) or above. However, natural signals collected from WSNs usually have relatively low information content as measured by the sparsity of their spectrum [12]. The theory of CS suggests that randomized low-rate sampling may provide an efficient alternative to high-rate uniform sampling by exploiting the prior of sparsity when recovering the original signals. The applications of CS in WSNs have been investigated extensively. The goal of applying CS on resource-constrained WSNs is to improve the accuracy of recovering the profile of the physical phenomena meanwhile reducing the amount of data transmitted through the network [6,13]. However, most of the WSNs monitor the temporal-spatial profile of the physical phenomena whose temporal and spatial spectrum can be sparse simultaneously while the current CS-based data collection methods in WSNs only consider the sparsity in either temporal or spatial dimension.

To further improve the performance of WSNs on energy consumption and signal reconstruction accuracy, we propose a new data collection strategy, CS^2^-collector, for WSNs based on the theory of two-dimensional CS (2DCS) by exploiting the two-Dimensional sparsity (2D-sparsity), i.e., the temporal and spatial sparsity, existing in most of WSNs. Like our evaluations on different types of real world sensors’ data, CS^2^-collector produces significant performance gain on signal reconstruction accuracy compared with the traditional one-dimensional CS (1DCS) based approaches with the same compression ratio or energy consumption budget. In other words, with the same goal of reconstruction accuracy, CS^2^-collector requires a significantly less amount of data transmitted through the network to the base station so that the energy consumption can be reduced.

The rest of this paper is organized as follows. Section 2 surveys the related work. Section 3 gives a brief introduction about CS. In Section 4, we describe the basic setup of network architecture and the CS^2^-collector in detail. The numerical simulations and real world dataset evaluations are presented in Section 5. In Section 6, we conclude the whole paper.

## 2. Related Work

In order to improve the efficiency of wireless sensor networks, numerous prior works have been done to investigate the availability of compressive sensing. The problem of energy consumption and data compression using CS is widely developed in the literature. Even though a rich literature focus on reconstruction algorithms and mathematical aspects, practical aspects and implementation problems have been developed lately.

The general problem of using CS in WSNs is investigated in several works, e.g. [14,15,16], and these papers focus on the problem of signal reconstruction. Ref. [14] evaluates the reconstruction accuracy by analyzing synthetic and real signals. Ref. [15] routes the measurement matrix to obtain a higher reconstruction quality, ref. [16] improves reconstruction accuracy by reordering input data to achieve a good compressive consequence. In [17], the authors apply one-bit compressive sensing in WSNs to reduce the number of measurements needed to transmit to the base station and propose a new optimisation algorithm to improve the reconstruction accuracy. Our work in this paper is different from papers above. Firstly, all of these papers assume that all the sensors sample and transmit the physical information at each time slot, but we proposed 2DCS in this paper, which show that some sensors do not transmit its data in a duration. Secondly, we exploit the data in temporal-spatial compression at the same time, and the outcomes of experiments show that 2DCS has a higher reconstruction precision.

Besides the classical digital implementation of CS used in all the papers above, in this paper, we also deal with CS when the signals are sampled at a sub-Nyquist frequency. As seen in previous sections, this compression technique is defined as analog CS, and the effects of analog compressive sensing architectures are discussed in [18].

Furthermore, the paper [19] uses a synthetic sparse matrix modifying the sampling rate to obtain a good reconstruction quality when energy consumption is smallest. The authors adjust the the sampling pattern to maintain a good reconstruction performance. Differently from other works, the authors do not focus on the issue of investigating different reconstruction algorithms, and they just exploit simple Basis Pursuit (BP) algorithm for reconstruction. Our work in this paper differs from this paper, and we try to exploit potential temporal-spatial correlations among nodes and increase the reconstruction quality by taking advantage of these correlations.

The sparse measurement matrices is investigated in [7,20], the energy consumption in a WSN is considered in [7], but there is no precise analysis on the energy for compression nor a relationship between energy consumption and reconstruction accuracy. In addition, the authors do not try to investigate potential temporal-spatial correlation in [20].

Ref. [21] reconstructs data collected using a sparse measurement matrix by exploiting a weighted form of the basis pursuit. However, the aim of the paper differs from ours: the paper [21] wants to obtain a acceptable reconstruction accuracy by presuming a well-defined frequency, but this pattern makes it easier to acquire better reconstruction accuracy. In addition, the probability that the reconstruction qualities are all good at different compressive ratios is tiny. While, in our work, we deal with the reconstruction without any prior knowledge about the information. Ref. [22] introduces the random access compressed sensing, which is a kind of low-rate CS, but the authors focus on investigating the network architecture more than exploiting CS for data compression.

Some recent work has been proposed to exploit the temporal-spatial correlation in WSNs to improve the performance of data collection [23,24,25,26,27,28,29]. Among them, [25] is the most related work, which exploits the joint sparsity model to reconstruct the temporal-spatial profile of WSNs, and we will compare it with our work in Section 5.3. The real world implementations are important for evaluation of practical performance of algorithms designed for WSNs. For example, the implementation of RSSI (Received Signal Strength Indicator) estimation algorithms in real world WSNs [30,31], background subtraction system on embedded camera networks [32,33] and wildlife recognition systems on acoustic sensor networks [34].

## 3. Introduction to Compressive Sensing

In order to make the paper self-contained, in this section, we provide a brief introduction for CS. CS is an emerging theory in signal processing. It aims to recover a high-dimensional sparse signal from a small number of measurements (or projections).

We consider a real-valued signal vector $x\in R^{n}$ in which the number of elements *n* is huge. To compress the signal, CS applies a projection matrix to reduce the dimensionality of the original signal as

(1) y=Φx,

where Φ is an m×n projection matrix and m≪n. Therefore, the projections vector $y\in R^{m}$ contains a significantly less number of elements than the original signal. According to the theory of CS, to achieve accurate reconstruction, the original signal *x* should be sparse in some transform domain, i.e.,

(2) x=Ψs,

where Ψ is some transform basis such as Discrete Cosine Transform basis, Wavelet Transform basis, etc. *s* is a sparse representation of the original signal *x* in transform basis Ψ. *s* is called *sparse* when only very few elements in *s* are non-zeros.

However, in most of the real applications, the ideal *sparsity* is not practical. It has been proved that the properties of CS still hold when the signals are *Compressible*. A signal *x* is said to be compressible if its representation *s* in transform basis Ψ only contains a very few number of dominant coefficients. More formally, descending the elements in *s* by its absolute value as ${\left|s\right|}_{\left(1\right)}\geq {\left|s\right|}_{\left(2\right)}\geq ...\geq {\left|s\right|}_{\left(n\right)}$, *x* is compressible if its representation satisfies

(3) $${\left|s\right|}_{\left(k\right)}\leq Ck^{-p}\;\forall k=1,2,...,n$$

for some p≥1 [35] and some constant C. As *sparsity* and *compressibility* produces the same properties in CS, in the rest of this paper, we will simply use *sparsity* instead of *compressibility* when concerning the practical applications to make the paper consistent.

According to the theory of CS, the original signal can be recovered by solving the following $\ell_{1}$ optimization problem,

(4) $$\hat{s}={\arg\min||s||}_{1}\;s.t.\;y=\Phi \Psi s$$

when the the projection matrix, i.e., Φ, satisfies the Restricted Isometry Condition (RIP) [36]. One striking result is that the random matrices generated from Gaussian or Bernoulli distributions satisfy the RIP condition.

## 4. CS${}^{2}$-Collector for Data Collection in WSNs

### 4.1. System Architecture

**Figure 1:**
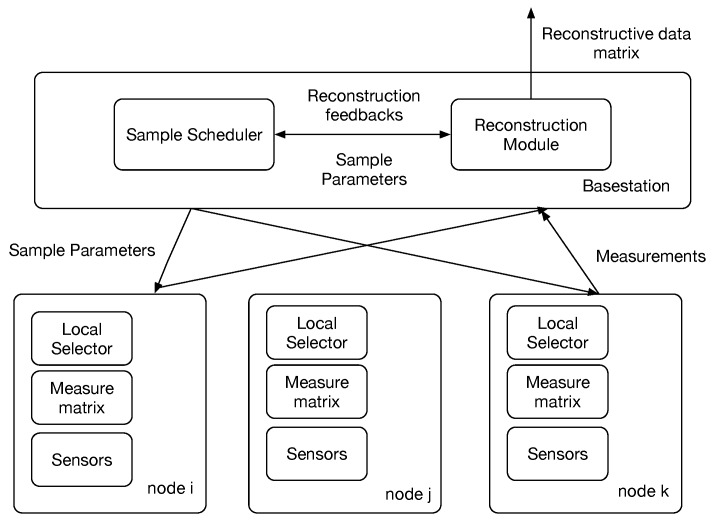
System architecture.

Figure 1 presents the system architecture of a typical WSN running CS${}^{2}$-collector to collect sensor data in an energy efficient way. In the base station, the sample scheduler decides the sampling parameters and some global control information like the compression ratio at local sensors, the schedule of time slots for each sensor to submit their compressed data and the random projection matrix used to compress the time-series data. Each local sensor compresses time series-data sensed and transmits the compressed data vector to the base station at its assigned time slots. When the base station receives compressed data vector from multiple sensors, it operates $\ell_{1}$ optimization to recover the original signals of the whole WSNs.

### 4.2. CS${}^{2}$-Collector

Data compression via compressive sensing is one of the popular choices to reduce the amount data required to transmitted through the network [14,15], so that the communication cost can be vastly saved.

Suppose a WSN consists of *N* sensors to monitor the temporal-spatial profile of some physical phenomena. According to the schedule from the base station, each sensor collects *M* data points during a time period of *T* seconds. Then, the temporal-spatial profile can be represented by a collection of data matrices $D\in R^{M\times N}$. The $i_{th}$ column of *D* is the data vector sensed by the $i_{th}$ sensor for *T* seconds. To exploit the temporal sparsity, each sensor applies CS locally to reduce the dimensionality of the time-series data vector. These operations, as a whole, can be uniformly represented by applying CS on the left of data matrix *D* which is, in math, left multiplying a random projection matrix $A\in R^{m_{t}\times N}$ with *D*,

(5) $$Y_{t}=AD,$$

where the random projection matrix *A* is randomly generated from Gaussian or Bernoulli distribution and $m_{t}\ll N$. The $i_{th}$ column of $Y_{t}$ is the compressed data vector from the $i_{th}$ sensor.

As spatial sparsity is also pervasive in WSNs, CS can be also applied to compress the sensor data in spatial domain:

(6) $$Y=ADB^{T},$$

where $B\in R^{m_{s}\times N}$ is a random projection matrix and $m_{s}\ll N$. *Y* is the compressed data matrix after 2DCS. Different from the Gaussian/ Bernoulli projection matrix *A*, the projection matrix *B* is a *sparse* random projection matrix. It is called *sparse* because only one entry at each row can be non-zero while each of its columns also contains only one non-zero. When the non-zeros are all **1** s, this operation is equal to randomly selecting $m_{s}$ sensors to submit their compressed data vector to base stations. It is known that, in the theory of CS, most of the information can be preserved by randomly choosing a small subset of the sensors. It has been proved in [8] that this sparse projection matrix satisfies the RIP condition. Because only a small subset of sensors are required to submit their data, the overall energy consumption during data transmission is reduced significantly.

### 4.3. Data Matrix Reconstruction at Base Station

When the base station receives the compressed data matrix *Y* from the end sensors, it starts reconstructing the original temporal-spatial profile of a physical phenomenon, i.e., the data matrix *D*.

#### 4.3.1. Two-Dimensional Sparsity

Similar to the traditional one-dimensional CS (1DCS) theory, the data matrix *D* can be accurately reconstructed after 2DCS if it is two-dimensional sparse (2D-sparse), i.e., it is temporally and spatially sparsely represented simultaneously. The 2D-sparse representation of the data matrix *D* can be expressed as

(7) $$D=PSQ^{T},$$

where $P\in R^{M\times M},Q\in R^{N\times N}$ are two transformation matrices in which the columns and the rows of *D* can be sparsely represented, respectively. *S* is 2D-sparse representation of *D*, whose entries are mostly zeros or close to zeros.

#### 4.3.2. Kronecker Product for $\ell_{1}$ Optimisation

The reconstruction of the data matrix can be converted to the one-dimensional data vector reconstruction problem by introducing Kronecker Product into the formulations of 2DCS. Then, it can be solved by standard $\ell_{1}$ optimization algorithms.

By applying Kronecker product, Equation (7) can be rewritten as,

(8) d=(P⊗Q)s,

where the operator ⊗ is the Kronecker product and d and s are two long vectors derived from vectorizing matrices D and S by column, respectively. The Kronecker product of *P* and *Q* produces an MN×MN block matrix expressed as

(9) $$P⊗Q=\left[\begin{array}{ccc} p_{11}Q & \cdots & p_{1M}Q\\ \vdots & \ddots & \vdots \\ p_{M1}Q & \cdots & p_{MM}Q \end{array}\right].$$

Similar to the transformation of 2D-sparsity, 2DCS in Equation (6) can be written as one-dimensional formation

(10) y=(A⊗B)d,

where A⊗B produces a $m_{t}m_{s}\times N^{2}$ projection matrix and $y\in R^{m_{t}m_{s}}$ is derived from concatenating the columns of the received compressed data matrix *Y*.

We define Ψ=P⊗Q as an equivalent basis where the temporal-spatial profile *D* is sparse and Φ=A⊗B as an equivalent projection matrix. With Equations (8), (9) and (10), 2DCS is transformed to a standard CS formation:

(11) y=Φd=ΦΨs;

therefore, the accurate and efficient reconstruction can be achieved by solving an $\ell_{1}$ optimization problem as below:

(12) $$\hat{s}={\arg\min||s||}_{1}\;s.t.\;y=\Phi \Psi s.$$

Then, the reconstructed long data vector $\hat{d}=\Psi \hat{s}$ and the estimated temporal-spatial profile $\hat{D}$ can be obtained by filling the columns of an M×N matrix with the entries in $\hat{d}$ in order. Again, in the real applications, the signals are normally *compressible* but not ideally sparse. However, the above statements of the 2DCS still hold when the signals are compressible.

## 5. Performance Evaluation

### 5.1. Goals, Metrics and Methodologies

The goals of our evaluations in this section is to demonstrate that (1) the temporal-spatial profile of some physical phenomena are 2D-sparse and (2) CS${}^{2}$-collector provides higher reconstruction accuracy than the 1DCS based approaches and the balanced temporal-spatial CS proposed in [25] based on the Joint Sparse Model under the same compression ratio.

The performance of CS${}^{2}$-collector is evaluated by both numerical simulations and real world datasets. We first use simulated sparse signals to provide some primary results in ideal conditions in numerical simulations. Then, in the dataset evaluation part, we use four different types of sensors’ data from the real world dataset to investigate the performance of CS${}^{2}$-collector under practical conditions. The dataset we use is from the Intel Berkeley Lab sensor network, which monitors an indoor environment of the Intel Berkeley Research Lab and provides four different types of sensors’ data, i.e., temperature, humidity, light and voltage. The software environment for the evaluations is MATLAB 2015b (MathWorks, Natick, MA, USA) and it runs on an Macbook pro laptop which features a 2.9 GHz Dual Core i5, 16GB memory and the operating system is Mac OS X EI Capitan (Apple, Cupertino, CA, USA).

Given that the spirit of CS${}^{2}$-collector is to use 2DCS to compress sensor data in both spatial and temporal domains, we consider comparing it with other two benchmark methods applying traditional 1DCS to compress the sensor data in spatial or temporal domain, respectively, and a most related state-of-the-art, which also exploits the temporal-spatial sparsity. We term CS in the spatial domain as Spatial 1DCS and CS in the temporal domain as Temporal 1DCS. In the Spatial 1DCS approach, the base station randomly selects a small subset of sensors to submit their sensor data at each time slot and applies $\ell_{1}$ optimization to reconstruct the full profile by exploiting the sparsity existing among different sensors adjacent to each other. While in Temporal 1DCS, each sensor data collects a long data vector, compresses it locally and transmits the compressed data vector to the base station. The base station reconstruct the compressed data vector from each sensor by exploiting the temporal sparsity existing in the time-series data collected consecutively. We also compare our work with the most related state-of-the-art, the balanced temporal-spatial CS proposed in [25]. They apply a different sparsity model, i.e., a Joint Sparsity Model, to improve the reconstruction accuracy of the temporal-spatial profile of WSNs. As it is different from our work by exploiting Joint Sparsity Model (JSM), we term it as JSM in the following statements. To make a fair comparison, the compression ratio for those three methods are identical during evaluations.

In this paper, we use the Mean Squared Error (MSE) as a performance metric to demonstrate the reconstruction accuracy. The MSE is defined as

(13) $$MSE=\frac{{\left||D-\hat{D}|\right|}_{F}^{2}}{MN},$$

where $\left|\right|\cdot {\left|\right|}_{F}$ is the Frobenius norm of the error matrix. We express the reconstruction accuracy of different data compression methods as MSEs under different compression ratios, where lower MSE stands for higher reconstruction accuracy achieved. The compression ratio is defined as

(14) $$\eta =\frac{\left(N-M\right)}{N},$$

where N is the number of elements in the original data matrix, and M is the number of elements in the compressed data matrix.

### 5.2. Numerical Simulations

**Figure 2:**
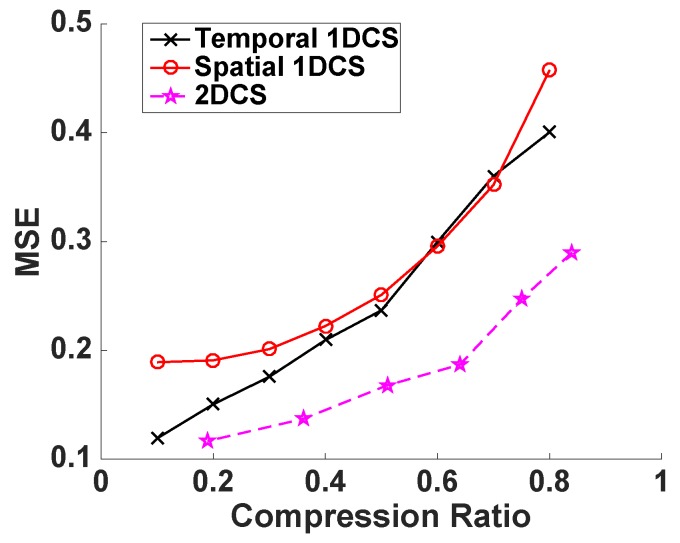
Performance of 2DCS and 1DCS on random sparse matrix with value of non-zero elements is 1.

We have proposed that CS${}^{2}$-collector is able to further compress the sensors’ data obtained from WSNs while accurately reconstructing the temporal-spatial profile of the physical phenomena, or, in other words, under the same compression ratio, CS${}^{2}$-collector is able to achieve higher reconstruction accuracy. In order to verify this proposition, we first evaluate the performance of CS${}^{2}$-collector, temporal 1DCS and spatial 1DCS under the ideal condition by generating a 50×50 sparse matrix whose non-zero elements are sparse in all columns and rows. The transform bases *P* and *Q* are DCT bases. Then, the simulated data matrix $D=PSQ^{T}$. In the data matrix *D*, a column stands for the time-series data from one sensor. To compress the data matrix, we randomly generate the Gaussian projection matrix *A* and the sparse projection matrix *B*. The compression ratio is determined by the number of rows in matrices *A* and *B*. We gradually change the compression ratio to evaluate the reconstruction accuracy of different data compression methods. From the results shown in Figure 2, we can observe that our proposed CS${}^{2}$-collector (based on 2DCS) produces significantly lower MSE compared with traditional 1DCS approaches, especially when the compression ratio is high. In plain words, CS${}^{2}$-collector can provide accurate reconstruction for the temporal-spatial profile of the physical phenomenon with significantly less amount of data transmitted to the base station, which results in energy efficiency and longer lifespan of the WSN.

### 5.3. Performance Evaluations on Real Dataset

#### 5.3.1. Intel Berkley Lab WSN Dataset

**Figure 3:**
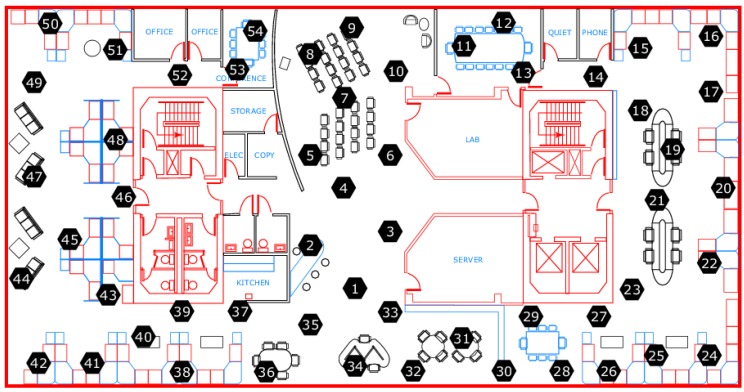
Intel Berkeley Lab sensor network.

Figure 3 shows a wireless sensor network composed of 54 sensors deployed at the Intel Berkeley Lab (Berkeley, CA, USA) monitoring temperature, humidity, lighting conditions of the surrounding environment as well as voltage of each sensor. Each sensor monitors and submits a package containing the above information once every 31 s. The dataset is available online. In this section, we evaluate the performance of our proposed CS${}^{2}$-collector on the four different types of sensors’ data in a practical dataset to provide more convincing evidence that the performance of our proposed method is singificantly better than traditional 1DCS based approaches and the JSM based approach.

#### 5.3.2. Dataset Preprocessing

The raw data in the Intel Berkley Lab dataset can not be directly used to evaluate the CS-based data compression methods as it is faulty and noisy. To reduce erroneous and missing data, we select 50 sensors to form the new WSN and split the dataset by every 50 time slots. Therefore, the size of each data matrix will be 50×50. Then, we remove the erroneous data and apply linear interpolation to fill in the missing data points to obtain a clean dataset. Then, we conduct performance evaluations on the four types of sensors’ data, respectively, and the results are as below.

#### 5.3.3. Temperature Data

We first evaluate the performance of CS${}^{2}$-collector on the data matrix obtained by temperature sensors in the Intel Berkley Lab WSN and compare it with two other 1DCS based approaches and JSM. The 1DCS benchmarks compress the sensor data in the temporal or spatial domains; therefore, they are termed as Temporal 1DCS and Spatial 1DCS, respectively. We first investigative the three types of sparsity of the data matrix, which are Temporal sparsity, Spatial sparsity and 2D-sparsity. We select DCT as the sparsifying basis, which is demonstrated to be a pervasively effective transform basis for most of the WSN sensors’ data [6,37].

**Figure 4:**
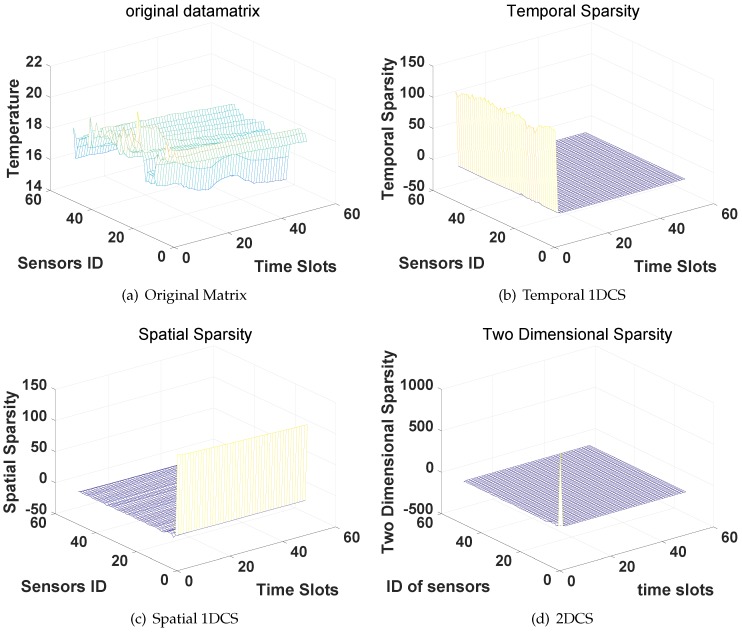
Sparsity of the temperature data matrix in DCT domain.

Figure 4 presents the original data matrix (Figure 4a), the temporal sparse coefficients (Figure 4b), spatial sparse coefficients (Figure 4c and 2D-sparse coefficients Figure 4d). As the results show in Figure 4b,c, we can find that the dominant elements (corresponding to the non-zeros in sparsity definition) are concentrated on the edge of the DCT coefficients matrices along the sensors ID (temporal sparsity) and the time slots (spatial sparsity), respectively. It indicates the sensor values collected from the same sensor (different time slots) or from the same time slot (different sensors) are both sparse in the DCT domain. Then, Figure 4d shows the distribution of 2D-sparsity where only few dominant elements concentrate at the corner of DCT coefficients matrix. Therefore, the original temperature data matrix collected from a WSN are 2D-sparse in the DCT domain.

**Figure 5:**
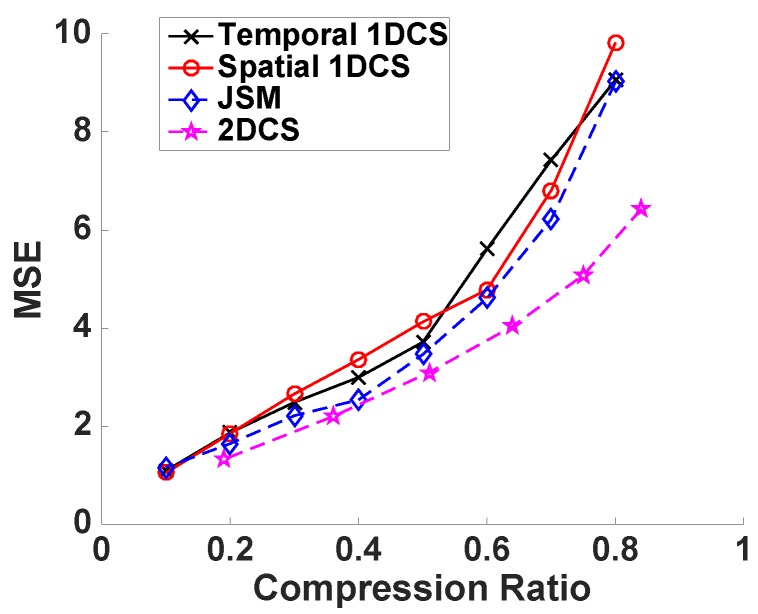
Reconstruction accuracy of CS-based approaches with temperature data.

After verifying the three types of sparsity of the temperature data matrix in the DCT domain, we compare the reconstruction accuracy of the four CS-based schemes. We apply $\ell_{1}$magic to solve the corresponding $\ell_{1}$ optimization problems because it has been compared with different state-of-the-art $\ell_{1}$ solvers in [38], and the results demonstrate that it provides the best reconstruction accuracy. We gradually change the compression ratio from 0.1 to 0.9 and use MSE as the performance evaluation metric. The trials are repeated 30 times and different random projection matrices are generated for each trial. Then, the average MSE over the 30 trials are computed, and the results are shown in Figure 5. We can observe from Figure 5 that CS${}^{2}$-collector, based on 2DCS, reduces the average MSE compared with the other two 1DCS approaches and JSM. The performance gain increases dramatically when the compression ratio is high. In other words, with the same goal of reconstruction accuracy at the base station, CS${}^{2}$-collector is able to reduce the data transmitted (corresponding to the high compression ratio) through the network so that the overall energy consumption can be reduced, and the lifespan of the WSN is increased.

#### 5.3.4. Humidity Data and Voltage Data

**Figure 6:**
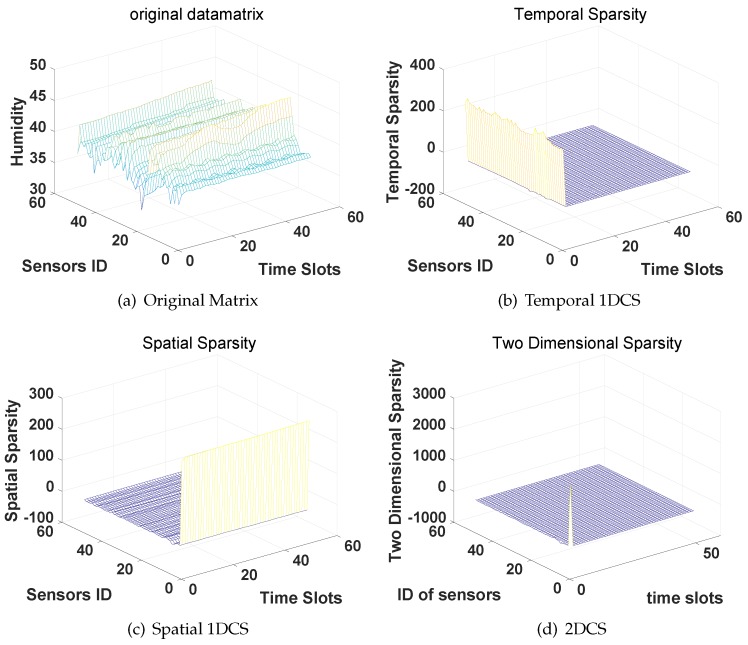
Compressibility of the humidity data matrix in the DCT domain.

**Figure 7:**
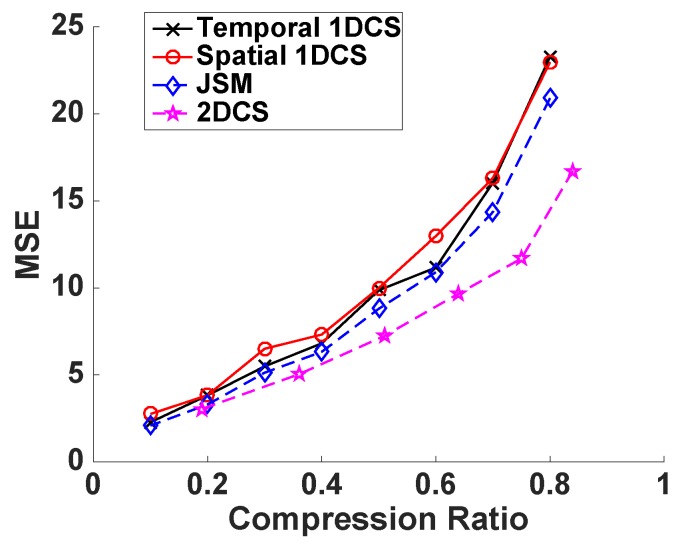
Reconstruction accuracy of CS-based approaches with humidity data.

**Figure 8:**
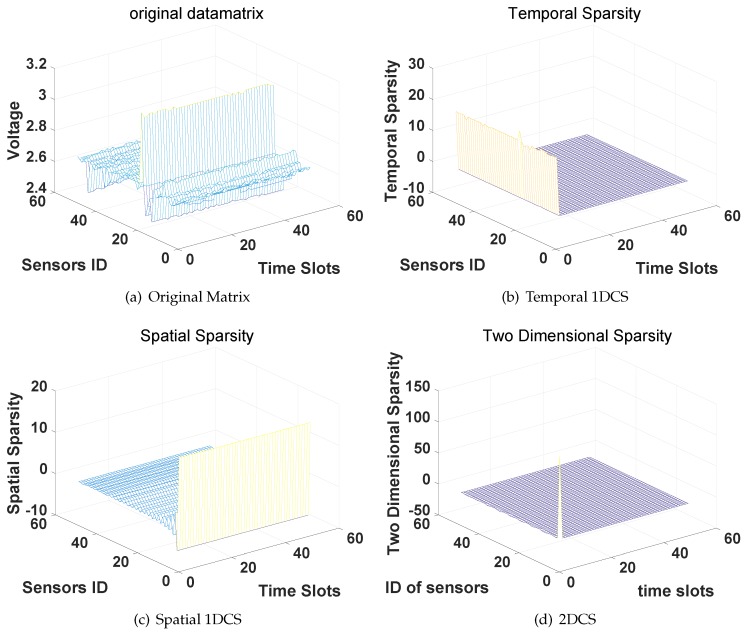
Compressibility of the voltage data matrix in the DCT domain.

**Figure 9:**
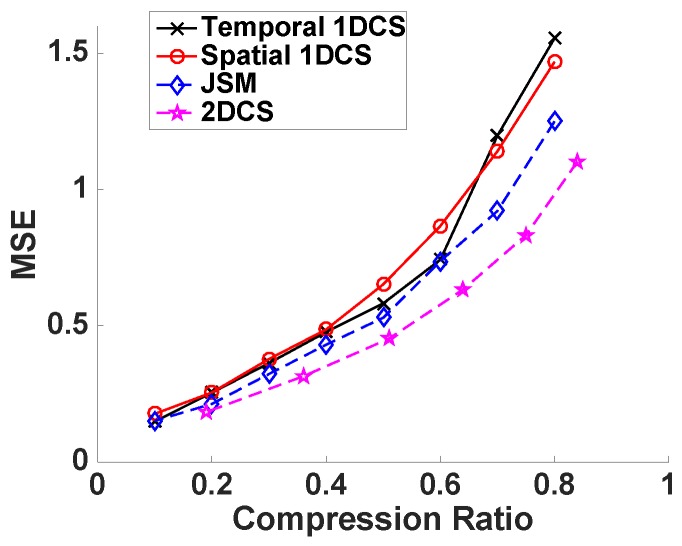
Reconstruction accuracy of CS-based approaches with voltage data.

We then evaluate the performance of the four CS-based approaches on the humidity data and voltage data. Again, we first examine the three types of sparsity of the sensors’ data in the DCT domain then evaluate the reconstruction accuracy of the four CS-based approaches. The results of the humidity are shown in Figure 6 and Figure 7 while the results of voltage data are shown in Figure 8 and Figure 9.

From the results we can find, similar to the results of the temperature data, the humidity data matrix and voltage data matrix are also temporal sparse, spatial sparse and 2D-sparse. In the evaluation of the reconstruction accuracy of the four CS-based approaches, we follow the same settings and steps as the evaluations on the temperature data. Again, CS${}^{2}$-collector achieves the highest reconstruction accuracy, and the performance gain is significant when the compression ratio is high.

#### 5.3.5. Lighting Data

**Figure 10:**
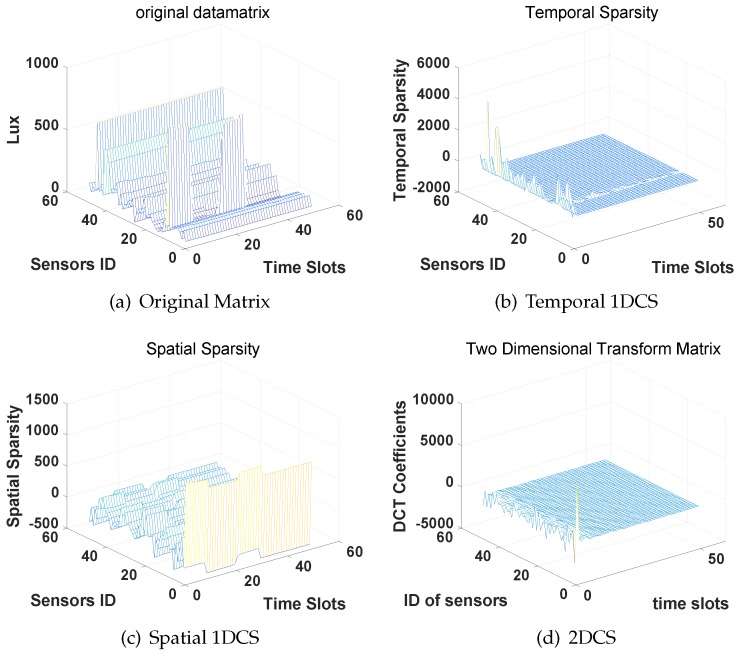
Compressibility of the light data matrix in the DCT domain.

Finally, we evaluate the performance of the CS${}^{2}$-collector on the data matrices obtained from lighting sensors. Different from the results from the other three types of sensors’ data, lighting data is not spatially sparse, therefore not 2D-sparse as shown in Figure 10c. This is because lighting is a kind of line-of-sight signal and is highly irregular and easily affected by artificial lighting; occlusions from the objects in the indoor environment will also influence the values of lighting data significantly even though the sensors are adjacently placed. Therefore, lighting sensors’ values are not spatially continuous.

**Figure 11:**
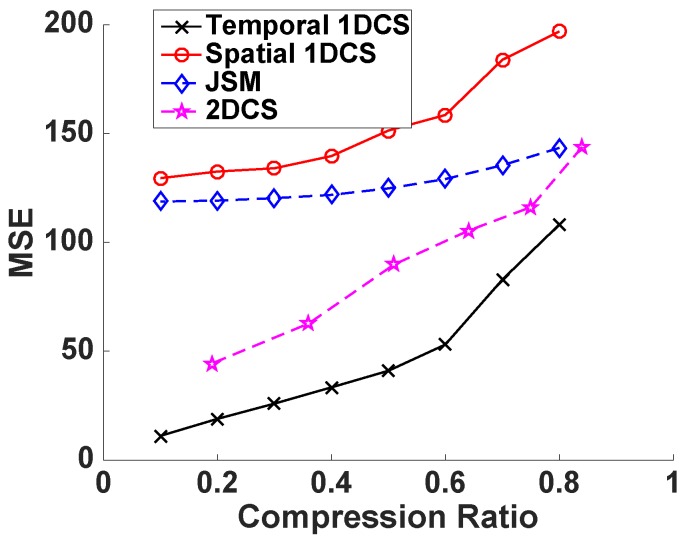
Reconstruction accuracy of CS-based approaches with lighting data.

The reconstruction accuracy results shown in Figure 11 also coincide with our observations from the sparsity evaluations: temporal 1DCS produces the lowest MSE because the lighting data is only temporally sparse, while our proposed CS${}^{2}$-collector and JSM are worse than temporal 1DCS but better than spatial 1DCS because the non-sparsity of the spatial dimension influences their performance.

Therefore, we should investigate the sparsity property of the sensors data before determining which data collection strategy should be adopted. This can be easily achieved by collecting some data samples and conducting some simulations beforehand. Then, the 2DCS based CS${}^{2}$-collector should be selected when the sensors’ data satisfies 2D-sparsity.

## 6. Conclusions

In this paper, we consider improving the tradeoff between the energy consumption and reconstruction accuracy of the WSNs. We propose a data collection strategy CS${}^{2}$-collector based on 2DCS to exploit the 2D-sparsity existing in the temporal-spatial profile of the physical phenomena monitored by most of the WSNs. According to the numerical simulations and real dataset evaluations, our proposed CS${}^{2}$-collector achieves significantly higher reconstruction accuracy than the traditional 1DCS based approaches and the JSM based approach, especially when the compression ratio is relatively high, which indicates that the overall energy consumption can be significantly reduced and a longer lifespan of WSNs are enabled by the CS${}^{2}$-collector.

In the future, we plan to improve the CS${}^{2}$-collector in two aspects. In the algorithm aspect, the JSM model can be incorporated in the 2DCS data collection scheme to further improve the reconstruction accuracy at a base station. Meanwhile, in the system aspect, we plan to implement our system on a testbed and evaluate its practical performance in real-world experimental environments.

## Figures and Tables

**Table 1:** The energy consumption load of humidity sensor.

Device	Duty Cycle	Average Current	The Ratio of Energy
Sensors	1.67%	9 (μA)	3.8%
Radio	1%	206 (μA)	86%
Microcontroller	0.4%	9.6 (μA)	4%
Quiescent	-	15 (μA)	6.2%
